# Retinal vascular changes after Silicon Oil removal in the Eye with Rhegmatogenous Retinal detachment

**DOI:** 10.1186/s40942-024-00587-9

**Published:** 2024-09-30

**Authors:** Ali Salehi, Mohammad Malekahmadi, Abolfazl Karimi, Afsaneh Naderi Beni

**Affiliations:** Isfahan Eye Research Center, Department of Ophthalmology, Isfahan University of Medical Sciences, Isfahan, Iran

**Keywords:** Optical coherence tomography angiography, Rhegmatogenous retinal detachment, Silicone oil removal

## Abstract

**Background:**

This study aims to examine vessel density changes in the optic nerve and macula following silicone oil removal (SOR) surgery in eyes with rhegmatogenous retinal detachment (RRD) at different time points by Optical Coherence Tomography Angiography (OCTA) in compared to the contralateral eye.

**Methods:**

A total of 43 eyes from 43 patients with silicone oil in their eyes for 3–9 months underwent OCT-A using AngioVue and optic disc-associated vessel density (VD) and thickness, macular-associated VD and thickness, Foveal avascular zone (FAZ) area, FAZ perimeter (PERIM), Acircularity index (AI), vessel density within a 300 μm wide region of the FAZ were compared between eyes. OCTA scans were performed one week before SOR and one month and three months after SOR.

**Results:**

The mean age of participants was 52.8 years (SD = 15.85) and a median visual acuity was 0.8 (range: 0.5-1.0). Notably, male participants constituted 67.4% of the sample. The preoperative mean value BCVA (logMAR) of patients was 0.73, and 3 months post-oil removal was 0.7727. Regarding optic disc parameters, RNFL thickness and vessel density (VD) measurements Peripapillary, whole disc, inside disc, and Disc Angio (superior, Nasal, inferior, temporal) did not change.

In analyzing macular thickness parameters, all of them (Whole and Fovea, parafoveal, and Perifovea) remained unchanged. Examining macular vessel density parameters revealed no significant changes across superficial and deep retinal layers. Finally, the comparison of the foveal avascular zone (FAZ) area and flow density (FD) parameters demonstrated consistent measurements with non-significant alterations observed in FAZ size (p = 0.6) and FD values (p = 0.49) over the monitored duration.

**Conclusion:**

There was no change in peripapillary VD and macular vessel density of the superficial capillary plexus (SCP) and deep capillary plexus (DCP) after silicone oil removal. FAZ and full retinal thickness  remained stable 3 month after SOR.

*Clinical trial number*: Not applicable.

## Introduction

Silicone oil (SO) is commonly used to treat various retinal illnesses such as rhegmatogenous retinal detachment and proliferative retinopathy. Silicone oil is utilized as a tamponade in cases of diabetic retinopathy, severe retinal damage, and endophthalmitis. Silicone oil can be utilized as an efficient intraocular tamponade because of its high viscosity and surface tension [1–3].

Several studies argue that this therapeutic procedure may have potential adverse effects. Complications including cataracts, keratopathy, glaucoma, and optic neuropathy have been documented after silicone oil injection [4–6].

Silicone oil tamponade might cause unexplained visual impairment as a secondary effect. Research utilizing Spectral-domain optical coherence tomography (SD-OCT) has demonstrated that silicone oil can induce alterations in the thickness of the inner retinal layer, potentially leading to visual impairment [7, 8].

Nowadays, Optical Coherence Tomography Angiography (OCTA) allows for non-invasive imaging of the retinal and choroidal vascular anatomy in three dimensions without the need for a contrast agent injection. The technique characterizes the microvascular and macrovascular circulation of the retina and optic nerve in patients with ocular vascular disorders [9]. Our study examined and contrasted alterations in superficial and deep macular vascular density and in patients who underwent pars plana vitrectomy with silicone oil removal using OCTA.

## Materials and methods

### Study design

The prospective cross-sectional study was conducted at the Department of Ophthalmology in Feiz Hospital, Isfahan, Iran between April and December 2023. The study protocol was accepted by the ethical committee of Isfahan University of Medical Sciences. All participants received written informed consent.

This study included 43 eyes from 43 participants with Unilateral RRD that were successfully repaired using pars plana vitrectomy (PPV) with light SO tamponade for 3–9 months and followed for more than 3 months after SOR surgery. These patients had a history of total RRD with up to grade 1 Proliferative Vitreoretinopathy without other ocular pathology. People with a history of glaucoma, ischemic optic neuropathy, or uveitis, or with systemic disorders such as hypertension or diabetes were not included in the study. Patients with a follow-up period of less than 3 months, the presence of recurrent retinal detachment, or simultaneous silicone oil removal surgery with epiretinal membrane (ERM) or internal limiting membrane (ILM) removal and had a history of ocular trauma or ocular surgery during follow-up period, were excluded from the study.

### Intervention

Patients who were candidates for silicone oil removal surgery at Feiz Hospital in Isfahan and met the criteria of our study were invited to participate in this study after giving detailed explanations. A complete medical record including past eye problems and general health was taken from each patient. Participants received various eye tests, including auto-refractometry, uncorrected and corrected distance visual acuity evaluation, slit lamp examination, Goldman and Tonopen tonometry for measuring intraocular pressure and dilated fundus examination with a 78-diopter lens. The evaluations took place one week before the silicone oil removal procedure and were repeated, one month, and three months after the operation. Macular and optic disc Optical Coherence Tomography Angiography (OCTA) scans were performed using the RTVue XR Avanti AngioVue system (Optovue Inc, Fremont, CA, USA) with AngioVue OCTA software by a single operator, one week before removing the silicone oil and after, one month, and three months after the surgery for all participants. All macular OCTA was performed by acquiring a 6 mm × 6 mm scan pattern which was automatically centered on the fovea. A three-dimensional projection artifact removal technique was applied to improve the accuracy of the data. PR-OCTA algorithms are designed to reduce projection artifacts by recognizing that the reflectance-normalized signal in these artifacts is weaker than the true in situ flow signal in the same axial line. The algorithm uses the structural OCT signal to weight the flow signal in the OCTA volume, keeping the highest in situ flow values and suppressing the rest to background levels. This process performed volumetrically, removes projection artifact tails from cross-sectional images, providing cleaner volumes compared to slab subtraction, though some limitations remain. All the investigated parameters were automatically calculated. The scans were evaluated by two ophthalmology specialists skilled in interpreting retinal OCT images. We analyzed the RNFL thickness, disc VD, macular (fovea, parafovea, perifovea) thickness, macular (fovea, parafovea, perifovea) superficial and deep capillary plexus (SCP and DCP) VD, FD and FAZ. All OCTA scans were reviewed to ensure their quality was sufficient (Scan Quality Indicator > 6/10). The same vitreoretinal surgeons performed SO (Oxane 5700 and 1300, Bausch & Lomb, Rochester, NY, USA) removal surgery. Fluid-air exchange was applied to reduce the number of emulsified SO droplets. The opposite eye was used as a control. The unaffected fellow eye was used in analyses for comparison with post-operative eyes.

### Statistical analysis

Data analysis utilized SPSS version 22 software. The statistical analysis employed descriptive statistics to characterize the patient.

 Comparative analyses, including repeated measures tests, assessed changes in measured parameters before and after silicone oil removal. Significance was determined at P-values of < 0.05.

## Results

The present research was conducted on 43 eyes with silicone oil removal and 43 healthy contralateral ones. The study investigated patient demographics and clinical characteristics, revealing a mean age of 52.8 years (SD = 15.85) and a median visual acuity of 0.8 (range: 0.5-1.0). Notably, male participants constituted 67.4% of the sample. Due to poor patient compliance and lack of regular visits, the follow-up period was determined to be 3 months. The basic characteristics of the patients are summarized in Table 1. Analysis of visual acuity measured in Log MAR units at baseline, month 1, and month 3 demonstrated a progressive increase in mean values from 0.7386 to 0.7727 over the three months. Regarding optic disc parameters before and after silicone oil tamponade removal, fluctuations in mean values were observed across various parameters from baseline to month 1 and month 3. Affected eyes had lower whole disc, and peripapillary vessel density compared to unaffected eyes in baseline (*p* < 0.05). Also affected eyes had lower superficial VD (whole, parafovea in temporal and inferior) and deep VD (parafovea in temporal and perifovea temporal) compared to unaffected eyes (*p* < 0.05). No statistically significant differences were found for FAZ or macular thickness ( foveal, parafoveal, and perifoveal) between the affected and contralateral healthy eyes. The results are shown in Tables 2, 3 and 4.

**Table 1:** Demographic and clinical characteristics of the patients

Investigated trait	Affected eyes	Unaffected eyes	*P* value (affected eyes vs. unaffected eyes(
Age(years)	52.8 ± 15.85 (15–92 ) Median :54		
Gender (male/female)	29/14 (67.4%/ 32.6%)		
Eye OD/OS	23/20	20/23	
Axial length	21.44 ± 1.3	22.02 ± 1.4	0.3
BCVA(log mar)	0.74 ± 0.17	0.13 ± 0.31	0.001
SSI	65.38 ± 10.5	69.15 ± 12.3	0.052
CDV ratio	0.23 ± 0.11	0.24 ± 0.27	0.9
CDH ratio	0.2 ± 0.15	0.2 ± 0.16	0.7
Rim area	1.95 ± 0.42	1.97 ± 0.44	0.4
Disc area	2.51 ± 0.46	2.43 ± 0.49	0.72

Best corrected visual acuity = BCVA Cup Disc Vertical = CDV Cup Disc Horizontal = CVH

**Table 2:** Comparison of circumpapillary retinal nerve fiber layer (RNFL) thickness and vessel density between affected eyes and unaffected eyes

	Affected eyes				Unaffected eyes			
Variable	Pre SO removal (A)	One month (B)	3 months (C)	Repeated p value	Baseline (D)	One month (E)	3 months (F)	Repeated p value
RNFL_1	105.32 ± 23.75	104.92 ± 23.09	105.97 ± 25.36	0.79	108.09 ± 20.37	104.95 ± 24.99	103.53 ± 22.59	0.24
Whole Disc VD	40.5 ± 6.82	40.08 ± 6.71	41.13 ± 7.12	0.1	43.64 ± 6.18	41.84 ± 7.85	41.56 ± 8.02	0.15
InsideDisc VD	43.47 ± 10.26	42.92 ± 10.41	44.49 ± 10.83	0.57	47.75 ± 9.38	45.69 ± 9.38	46.2 ± 9.92	0.42
Peripapillary VD	42.55 ± 7.82	42.87 ± 8.01	44.28 ± 7.64	0.1	47.33 ± 7.02	45.3 ± 8.59	44.718.82	0.27
Superior disc VD	47.11 ± 7.76	46.92 ± 7.96	47.68 ± 6.29	0.9	45.74 ± 9.79	44.04 ± 10.07	44.24 ± 10.25	0.48
Nasal disc VD	38.51 ± 8.696	39.47 ± 9.27	41 ± 9.02	0.06	42.27 ± 9.36	41.16 ± 9.26	41.38 ± 9.79	0.45
Inferior disc VD	42.12 ± 9.70	42.4 ± 9.88	43 ± 9.87	0.81	47.87 ± 9.59	48 ± 9.43	46.88 ± 10.13	0.45
Temporal disc VD	41.84 ± 9.53	41.76 ± 9.40	41.92 ± 10.29	0.45	47.54 ± 9.57	46.1 ± 10.68	44.9 ± 10.75	0.2

	*P* value A and D	*P* value C and F	*P* value A and B	*P* value A and C
RNFL_1	0.59	0.663	0.86	0.25
Whole Disc VD	0.03	0.797	0.27	0.26
Inside Disc VD	0.05	0.454	0.69	0.82
Peripapillary VD	0.009	0.823	0.36	0.54
Superior disc VD	0.5	0.083	0.76	0.38
Nasal disc VD	0.07	0.863	0.24	0.33
Inferior disc VD	0.01	0.1	0.59	0.87
Temporal disc VD	0.01	0.23	0.28	0.13

Vessel density = VD

**Table 3:** Comparison of macular thickness between affected eyes and unaffected eyes

	Affected eyes				Unaffected eyes			
	Pre SO removal (A)	One month (B)	3 months (C)	Repeated p value	Baseline (D)	One month (E)	3 months (F)	Repeated p value
Whole macular thickness	330.6 ± 154.96	328.1 ± 143.93	310.8 ± 124.68	0.46	286.1 ± 60.80	292.7 ± 75.93	309.2 ± 111.89	0.27
Foveal thickness	318.5 ± 161.17	326.1 ± 145.54	306.3 ± 112.24	0.51	267.3 ± 75.75	271.02 ± 77.62	277.8 ± 88.52	0.41
Parafoveal thickness	323.8 ± 154.59	330.5 ± 145.39	314.1 ± 113.04	0.67	306.7 ± 101.24	305.1 ± 112.80	310.7 ± 120.17	0.71
Perifoveal thickness	324 ± 155.19	326.2 ± 154.87	308.7 ± 130.06	0.52	289.9 ± 76.78	288 ± 94.11	303.02 ± 121.57	0.49

	*P* value A and D	*P* value C and F	*P* value A and B	*P* value A and C
Whole macular thickness	0.09	0.9	0.87	0.88
Foveal thickness	0.06	0.2	0.71	0.94
Parafoveal thickness	0.55	0.85	0.72	0.86
Perifoveal thickness	0.22	0.8	0.89	0.94

**Table 4:** Comparison of macular vessel density  between affected eyes and unaffected eyes

	Affected eyes				Unaffected eyes			
	Pre SO removal(A)	One month (B)	3 months (C)	Repeated p value	Baseline (D)	One month (E)	3 months (F)	Repeated p value
Superficial VD								
Whole	38.66 ± 10.02	36.04 ± 13.14	37.44 ± 11.60	0.41	43.78 ± 9.541	43.18 ± 10.34	43.42 ± 11.54	0.89
Fovea	30.87 ± 15.09	27.71 ± 13.63	28.39 ± 13.25	0.07	30.71 ± 11.71	29.87 ± 11.39	32.13 ± 11.81	0.17
Parafovea	37.76 ± 11.42	37.81 ± 11.15	37.64 ± 11.31	0.05	45.23 ± 10.90	43.41 ± 10.28	44.44 ± 10.34	0.1
Temporal	37.47 ± 11.79	37.2 ± 13.65	37.12 ± 11.62	0.92	46.76 ± 11.00	44.52 ± 11.44	43.76 ± 12.65	0.42
Superior	42.36 ± 11.89	38.1 ± 15.87	39.27 ± 14.18	0.12	44 ± 12.001	42.29 ± 12.30	42.94 ± 12.62	0.23
Nasal	39.21 ± 13.73	38.93 ± 12.93	40.36 ± 12.72	0.81	44.4 ± 13.04	43.32 ± 13.37	45.08 ± 14.04	0.12
Inferior	38.49 ± 13.02	38.72 ± 13.19	37.71 ± 12.25	0.73	46.37 ± 12.24	43.65 ± 11.96	43.95 ± 12.78	0.1
Perifovea	42.82 ± 8.628	41.72 ± 9	42.21 ± 8.76	0.79	45.41 ± 8.49	45.73 ± 8.02	46.64 ± 8.51	0.35
Temporal	39.8 ± 11.152	37.18 ± 10.12	37.43 ± 10.13	0.3	43.57 ± 8.91	42.47 ± 9.35	42.41 ± 8.98	0.48
Superior	43.89 ± 9.859	42.87 ± 12.92	44.61 ± 10.14	0.85	46.81 ± 9.02	47.32 ± 8.68	47.61 ± 8.99	0.72
Nasal	47.07 ± 10.41	45.92 ± 12.09	48.77 ± 9.67	0.3	49.73 ± 9.25	49.18 ± 8.88	50.5 ± 10.1	0.1
Inferior	43.67 ± 8.49	38.34 ± 15.43	40.43 ± 13.54	0.23	46.4 ± 8.89	46.45 ± 9.17	47.2 ± 9.914	0.46
Deep VD								
Whole	37.68 ± 10.20	35.46 ± 10.44	36.83 ± 9.93	0.36	41.48 ± 10.77	40.38 ± 11.13	41.2 ± 11.001	0.24
Fovea	37.49 ± 16.83	33.98 ± 16.03	37.25 ± 14.69	0.3	37.77 ± 13.18	35.37 ± 14.15	36.97 ± 15.13	0.19
Parafovea	41.36 ± 11.38	38.32 ± 12.56	40.7 ± 11.40	0.35	44.86 ± 11.24	43.44 ± 12.57	45.18 ± 12.37	0.3
Temporal	37.55 ± 14.05	36.89 ± 14.86	39.1 ± 13.15	0.68	45.4 ± 13.57	45.88 ± 13.16	46.97 ± 13.08	0.25
Superior	44.58 ± 12.96	41.36 ± 15.06	44.18 ± 12.71	0.48	45.69 ± 8.85	44.06 ± 10.84	45.82 ± 11.56	0.22
Nasal	41.59 ± 15.62	36.3 ± 17.17	38.98 ± 16.96	0.06	44.73 ± 14.51	43.79 ± 14.83	46.63 ± 14.03	0.15
Inferior	42.52 ± 14.56	38.5 ± 17.52	37.4 ± 17.76	0.11	45.35 ± 10.85	42.98 ± 12.42	43.21 ± 14.49	0.37
Perifovea	38.43 ± 11.63	36.41 ± 11.53	36.5 ± 12.73	0.26	42.65 ± 11.33	40.79 ± 11.51	41.89 ± 11.23	0.24
Temporal	34 ± 15.23	31.43 ± 13.92	34.09 ± 13.82	0.35	43.58 ± 13.38	41.84 ± 13.17	41.59 ± 14.03	0.28
Superior	39.68 ± 13.45	37.01 ± 15.41	39.49 ± 14.37	0.5	43.97 ± 10.68	43.77 ± 10.00	45.01 ± 10.44	0.26
Nasal	40.44 ± 13.55	37.85 ± 13.34	38.62 ± 12.49	0.72	42.24 ± 10.67	40.48 ± 13.56	42.27 ± 12.70	0.3
Inferior	38.77 ± 11.19	36.87 ± 11.30	37.17 ± 10.53	0.61	42.26 ± 12.05	40.71 ± 12.15	41.68 ± 12.29	0.43
FAZ1	0.89 ± 2.22	0.87 ± 2.21	0.82 ± 2.16	0.6	0.5656 ± 1.19	0.58 ± 1.19	0.60 ± 1.22	0.75
PERIM	3.25 ± 4.304	3.19 ± 4.32	3.04 ± 4.19	0.37	2.73 ± 3.02	2.80 ± 3.02	2.86 ± 3.1	0.69
FD	38.13 ± 16.34	38.64 ± 13.36	38.13 ± 13.48	0.49	46.88 ± 11.55	43.67 ± 12.93	44.66 ± 13.62	0.2

	*P* value A and D	*P* value C and F	*P* value A and B	*P* value A and C
Superficial VD				
Whole	0.02	0.02	0.19	0.41
Fovea	0.95	0.2	0.41	0.21
Parafovea	0.004	0.008	0.6	0.94
Temporal	0.001	0.01	0.94	0.7
Superior	0.54	0.23	0.04	0.1
Nasal	0.08	0.12	0.91	0.73
Inferior	0.007	0.03	0.49	0.71
Perifovea	0.18	0.02	0.49	0.65
Temporal	0.23	0.03	0.49	0.15
Superior	0.1	0.19	0.04	0.83
Nasal	0.25	0.47	0.38	0.22
Inferior	0.19	0.02	0.08	0.25
Deep VD				
Whole	0.11	0.07	0.15	0.9
Fovea	0.93	0.93	0.13	0.85
Parafovea	0.11	0.1	0.13	0.68
Temporal	0.01	0.01	0.48	0.55
Superior	0.66	0.55	0.04	0.71
Nasal	0.36	0.03	0.91	0.13
Inferior	0.33	0.11	0.27	0.03
Perifovea	0.11	0.05	0.12	0.31
Temporal	0.005	0.02	0.12	0.95
Superior	0.13	0.06	0.27	0.65
Nasal	0.53	0.23	0.71	0.22
Inferior	0.22	0.1	0.3	0.97
FAZ1	0.44	0.61	0.07	0.11
PERIM	0.56	0.83	0.45	0.15
FD	0.01	0.05	0.92	0.76

vessel density=VD

**Fig. 1:**
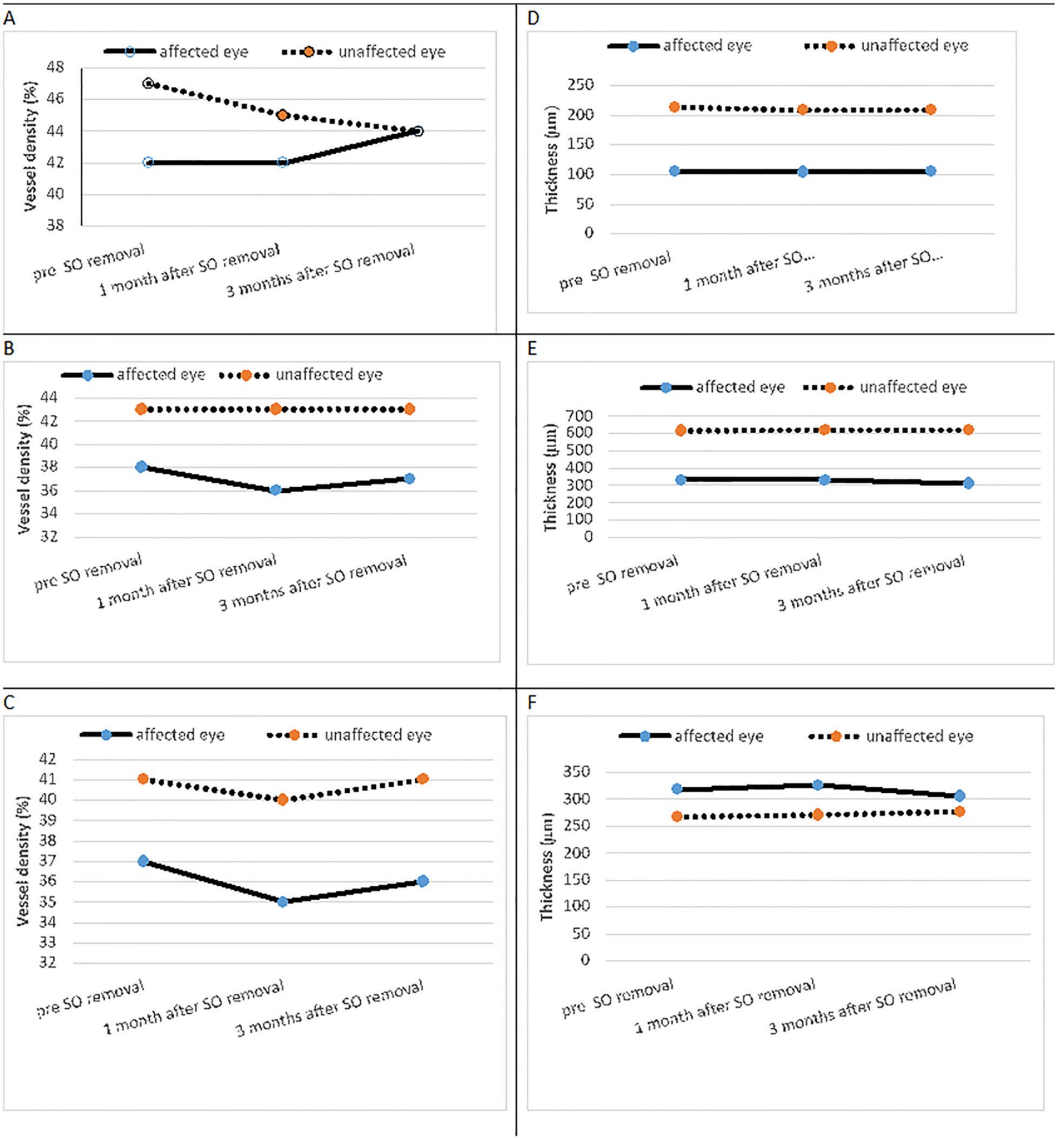
Changes in retinal vessel density and thickness before and after surgery in affected and unaffected eyes. As shown in figure, no significant changes occurred after so removal. **A** Peripapillary vessel density at optic nerve head   **B** Superficial  macular vessel density **C** Deep macular vessel density **D** Peripapillary RNFL    **E** Total macular thickness  **F **Total  Foveal thickness

The repeated escarpment tests for the affected eyes and healthy eyes revealed no statistically significant changes over time for the following measurements: RNFL, and RPC density (whole, inside, peripapillary -superior, temporal, nasal, inferior), superficial and deep macular vessel density and macular thickness. In the case of deep vessel density, significant mean differences were observed in the parafovea superior region between preoperative time and one month after silicone oil tamponade removal time (mean difference = −1 μm, *p* = 0.04) and in the parafovea inferior region between the preoperative and three months after silicone oil tamponade removal (mean difference = −5 μm, *p* = 0.03). Finally, the comparison of the foveal avascular zone (FAZ) and flow density (FD) parameters before and after silicone oil tamponade removal demonstrated consistent measurements with non-significant alterations observed in FAZ size (*p* = 0.6) and FD values (*p* = 0.49) over the monitored duration. Changes in retinal vessel density and thickness before and after surgery in affected and unaffected eyes are shown in Fig. 1.

## Discussion

Silicone oils are commonly used in vitreoretinal surgery due to their long-lasting properties, biocompatibility, and inertness to chemicals [11]. These attributes help maintain the connection between the retina and the retinal pigment epithelium, blocking harmful substances from the healing area, and making them essential in such operations [12]. However, because silicone oils are less dense than water, they float in the eye, potentially providing insufficient support for the lower retina if the vitreous cavity isn’t filled [13]. Silicone oils can emulsify and disperse, leading to complications like inflammation, vision changes, increased intraocular pressure, and glaucoma, often necessitating removal surgery. Despite removal, tiny droplets can persist for up to 11 years, though about 30% of patients see improved visual acuity post-removal [14].

Optical Coherence Tomography Angiography (OCTA) revolutionizes optic nerve and macula evaluation by offering precise, non-invasive imaging of retinal blood vessels, aiding in early disorder detection and treatment decisions with high-resolution images without the need for contrast chemicals [15]. In our study, we focused on evaluating the optic nerve and macula, finding no statistically significant changes over time for the optic disc and macular parameters in OCT angiography, consistent with previous studies [10, 16]. Xiang et al. [10] and Nassar GA [16]. showed that SO use for less than six months has no significant effect on the macular capillary vessel density (VD) of the superficial capillary plexus (SCP) and deep capillary plexus (DCP). However, we observed significantly lower peripapillary optic disc and superficial and deep macular vessel densities in silicone oil removal eyes compared to healthy eyes, with no significant changes over three months. Previous studies regarding the analysis of OCTA findings in SO removal are summarized in Table 5 [10, 16–23].

**Table 5:** Summery of recent studies of silicone oil removal effect retina

Author	Number	Time	Oct	Results
WU XIANG (10)	20 patients	OCTA examination 1 week previously, 3 months after SOR.	OCTA (RTVue-XR Avanti; Optovue	After silicone oil tamponade (SOT), macular capillary vessel density and full retinal thickness remain stable, but the inner retina may become compressed and thinner over six months.
Kheir WJ (17)	Ten eyes of 9 patients	OCTA examination before during, 3 months after SO tamponade	OCT(e (Cirrus HD-OCT; Carl Zeiss Meditec, Dublin, California)	Silicone oil (SO) tamponade caused foveal flattening and thinning of the inner retinal layers, which were reversible upon SO removal. Outer retinal thickness remained unchanged.
Karasu B (18)	70 eyes of 70 patients with	MT and SFCT 1 day before 3 months after SO removal	(SD-OCT) (Heidelberg Engineering, Heidelberg, Germany	After silicone oil removal, macular and choroidal thicknesses significantly decreased, and prolonged nine-month SO tamponade was linked to subfoveal choroidal thinning and reduced visual acuity after retinal detachment surgery.
Liu Y (19)	33 patients: 16 eyes : gas tamponade 17 eyes :silicone oil tamponade.	Mean follow-up duration was 36.1±3.6 months	Spectral domain OCT system (software Version 2017.1.0.155; RTVue-XR Avanti, Optovue Inc., Fremont, CA)	Eyes in the silicone oil tamponade group had worse visual acuity, lower vessel densities, and thinner inner retinal thickness compared to the gas tamponade group after vitrectomy for macular-on retinal detachment.
Lee J (20)	30 patients	Six months after SO removal	SS-OCT( DRI OCT-1 Atlantis; Topcon Corporation, Tokyo, Japan)	Silicone oil tamponade and removal for retinal detachment changed retinal and peripapillary thickness but did not significantly affect vessel density on OCTA
Prasuhn M (21)	19 patient	OCTA examination before and four weeks after removal of SO.	OCTA(Zeiss Cirrus HD-OCT (AngioPlex, CIRRUS HD-OCT model 5000, Carl Zeiss Meditec, Inc., Dublin, CA, USA)	Silicone oil (SO) removal impacts choroidal perfusion and causes shifts within choroidal sublayers. Further studies are needed to understand these effects better.
Hou Y (22)	Fifty eyes	OCTA examination at 1 day, 7 days, 1 month, and 3 months after removal of SO	OCTA Zeiss HD-OCT 5000 with AngioPlex; Carl Zeiss Meditec, Oberkochen, Germany)	Silicone oil (SO) tamponade reduces superficial and deep vascular densities (SVD and SPD), which increase after SO removal in the macular region.
Karakosta C (23)	28 patients	Week and 1, 3, 6, and 12 months postoperatively.	OCTA((RTVue-XR Avanti, AngioVue, Optovue)	Improvements are significant from the first postoperative week to 6 and 12 months, suggesting potential benefits for best corrected visual acuity (BCVA) up to one year after SO removal.
Nassar GA (16)	30 eyes subjected	OCTA examination preoperatively and 1 month following SO remova	OCTA Avanti RTVue system (Optovue Inc, Fremont, CA, USA).	SO removal improved retinal sensitivity, vision, and optic nerve perfusion, but did not significantly affect macular vessel density (VD)

In the current study, the average total retinal thickness (whole, fovea, parafovea, and perifovea) was higher in eyes with silicone oil compared to healthy eyes but not significantly so, which aligns with a previous study [23]. There was no significant change in average total retinal thickness after silicone oil removal, consistent with prior findings [23]. We noted a decrease in the fovea, parafovea, and perifovea thickness measurements between baseline and three months post-operation, but these changes were not significant. This is the first study to separately evaluate these macular regions. After silicone oil removal macular thickness(whole, fovea, parafovea, perifovea) was not significantly different over three months which is consistent with Karakosta C [23]. 

The findings showed no significant changes in superficial and deep macular vessel density pre- and post-silicone removal. Prior studies demonstrated significant reductions in SCP and DCP densities in individuals after SO removal [10, 16, 20], but other reports indicated changes in vessel density [16, 23]. In our study, optic disc vessel densities decreased over time in the control group, but increased in the patient group but not significantly.

Our study observed a greater size and perimeter of the foveal avascular zone (FAZ) in the SO group compared to the control group, suggesting higher vascular impairment, though insignificant. FAZ parameters remained stable across different groups. These findings of our study were consistent with previous studies [10, 16, 19, 23] The FAZ parameters (area, perim, and FD) remained stable across different groups.

Our studies indicate that peripapillary vessel density increased over time in affected eyes, similar to findings by Karakosta C and Hue Y [22, 23]. , but. Nassar G A [16]reported a significant increase in postoperative VD of the whole disc and peripapillary capillary plexus. Our study showed different trends over time in control versus patient eyes. Vessel density decreased over time in the control group but increased in the patient group. These results collectively suggest that patients experienced an increase in vessel density over time, contrasting with the control group where a decrease was observed.

Lee et al. [24]found that SO tampon duration significantly correlates with VD in the DCP. Discrepancies between our findings and others may be due to differences in SO tamponade durations, inclusion and exclusion criteria, OCTA devices, sample sizes, follow-up periods, and study designs. Our study had several limitations: the cross-sectional methodology, small sample size, short duration of follow-up, and lack of investigation into systemic medication effects. Prospective studies with larger sample sizes could provide more insights into the effect of SO on OCT angiography.

## Conclusion

Silicone oils are essential in vitreoretinal surgery for their longevity, biocompatibility, and chemical inertness, but they can float in the eye and cause complications like inflammation and increased intraocular pressure. Our study found no significant changes in retinal thickness or vessel density over time after silicone oil removal, with observed trends consistent with previous research.

## Data Availability

No datasets were generated or analysed during the current study.
